# Comparative analysis of classical growth models and artificial neural networks in predicting egg production parameters in three commercial broiler parent stocks

**DOI:** 10.1016/j.psj.2025.106356

**Published:** 2025-12-28

**Authors:** Zahra Moradi Gharajeh, Hassan Darmani Kuhi, Navid Ghavi Hossein-Zadeh

**Affiliations:** Department of Animal Science, Faculty of Agricultural Sciences, University of Guilan, Rasht, Iran

**Keywords:** Egg production, Artificial neural network (ANN), Growth model, Broiler parent stock, Prediction accuracy

## Abstract

In the rapidly evolving poultry industry, effective management is dependent on precise forecasting of production metrics. As producers seek to maximize yield while minimizing costs, the ability to accurately predict egg production parameters becomes essential. Traditional growth models have long been employed for this purpose, but with advancements in computational techniques, artificial neural networks (ANNs) present a promising alternative. This paper examines the efficacy of various classical growth models, Gamma, compartmental, logistic, curvilinear Gompertz, Richards, and Morgan models, alongside a multi-layer feed-forward ANN model in predicting weekly and cumulative production metrics of three distinct commercial broiler parent stock (Ross, Arbor Acres, and Cobb). The analysis revealed that the Morgan model performed as the most accurate for modeling cumulative data, showcasing a superior fit characterized by high adjusted R² values and low RMSE, AIC, and BIC. In contrast, the Logistic - curvilinear model demonstrated high precision for weekly data. The ANN significantly surpassed the best traditional Logistic curvilinear and Morgan models in predicting weekly and cumulative production metrics based on RMSE, AIC, and BIC criteria (RMSE = 100% both for weekly and cumulative productive metrics, AIC = 91.6% for weekly and 58.3% cumulative productive metrics, and BIC = 66.6% both for weekly and cumulative productive metrics). Although the findings underscore the strength of the classical growth model for cumulative data forecasts in poultry production systems, the advantages of ANNs in the prediction of egg production metrics represent a vital advancement toward more responsive flock management strategies. By integrating classical growth models with ANN approaches, poultry producers can enhance decision-making processes, leading to improved productivity and sustainability. The integration of these methodologies provides a powerful framework for accurate forecasting, ultimately advancing the poultry industry's capacity for efficient production management.

## Introduction

Effective management of egg production in layer breeder strains is crucial for optimizing production and ensuring sustainable productivity in the poultry industry (Ghazanfari et al., 2011). Accurate predictions of egg mass, hatching egg production, and overall egg production over time can assist in improving resource allocation, flock management, and profitability by increasing revenue, decreasing costs, or a combination of both. Understanding the egg production curve in broiler breeder hens is key to developing effective nutritional strategies and enhancing management practices. Growth models and artificial intelligence-based approaches, such as artificial neural networks (ANN), provide powerful tools for accurately predicting these properties, contributing to better decision-making and overall performance improvement in poultry operations (Faraji-Arough et al., 2023).

Hatchability is one of the key indicators in poultry production and is highly influenced by egg quality and weight. Various studies have shown that larger eggs generally have higher hatchability rates, and the chicks hatched from them tend to have greater body weights (Alabi et al., 2012a, 2012b). Since egg size is directly related to embryonic growth, residual yolk content, and chick quality, paying attention to this factor can significantly improve the economic efficiency of poultry farms. An increase in egg weight not only enhances hatchability rates but also leads to the production of stronger and healthier chicks, thereby reducing post-hatch mortality (Farooq et al., 2003). Egg weight is a critical trait for assessing both the quality and grading of eggs and is measurable without breaking the shell (Wilson and Suarez, 1993; Farooq et al., 2001). The proportions of internal components (albumen, yolk, and shell) are directly related to the egg's weight, and these proportions can vary significantly depending on the hen’s strain (Pandey et al., 1986). Additionally, the age of the hen plays a vital role in determining egg weight, with a general trend of increasing egg weight as the hen matures, eventually stabilizing toward the end of the laying cycle (Moreno et al., 2024). This indicates that egg weight should be considered not only as a quality indicator but also as a biologically relevant factor in the productive performance of laying hens**.**

Egg mass is a key factor in reproductive success in poultry, directly influencing hatchability, chick weight, and early growth. Larger eggs are generally associated with higher hatchability rates and heavier, stronger chicks (Krist, 2011; Kumar et al., 2024). This characteristic is also linked to incubation duration, embryonic metabolism, and hatchling size at the time of hatching (Rahn and Ar, 1974; Deeming and Birchard, 2007). From an evolutionary perspective, egg mass represents a trade-off between offspring quality and quantity, and its relationship with body size varies depending on the physiological and ecological traits of the species (Deeming, 2007).

Egg production is defined as the number of eggs laid within a specific period or cumulatively, and mathematical models are essential for describing the laying curve and assessing egg production across different periods (Narinc et al., 2013). There is limited research focusing on modeling egg production in breeder hens due to the longer duration required to track egg production (Narinc et al., 2014).

The egg production curve in hens is similar to the lactation curve in cows (Wood, 1967), and the functions used to describe the lactation curve have also been adapted for modeling egg production (Wolc et al., 2015). These models typically include biologically significant parameters that help in understanding the egg production process (Savegnago et al., 2011).

The Gamma model is the most widely used due to its simplicity and good fit with the data, though compartmental and Logistic-curvilinear models are also employed (Wood, 1967; Gavora et al., 1982; Narinc et al., 2013). The application of appropriate mathematical models is crucial for predicting egg production and making economic decisions in the poultry industry (Aggrey, 2002; Savegnago et al., 2011).

Productive performance in broiler breeder hens is influenced by a complex interaction of genetic, physiological, nutritional, and environmental factors. Understanding and predicting these biological responses are essential for improving management strategies and maximizing production efficiency. While traditional growth models effectively describe the overall production trend, they are limited in capturing the nonlinear and dynamic interactions inherent in biological systems (van der Klein et al., 2020). The inherent variability and interdependence among productive traits highlight the need for advanced modeling approaches capable of capturing and learning complex relationships within biological data. In this context, ANN offer a flexible and powerful alternative, as they can model nonlinear patterns, adapt to complex biological behaviors, and provide more accurate predictions without requiring predefined mathematical assumptions (Ghazanfari et al., 2011). Therefore, integrating ANN with traditional growth models can enhance our understanding of production dynamics and support more precise decision-making in broiler breeder breeding and management programs. Establishing this link between biological principles and model-based prediction is crucial for developing strategies to optimize nutrient use, improve growth performance, and support genetic improvement programs.

Despite many advantages, traditional models have limitations in capturing complex relationships in the data, whereas ANN have demonstrated the ability to model nonlinear relationships and complex patterns in agricultural settings, improving prediction accuracy without explicit assumptions (Zhang et al., 2002). The ANN is an advanced method capable of simulating complex nonlinear relationships between inputs and outputs. Key advantages of ANN include their adaptability to changing inputs and outputs, fault tolerance, and interpolation capabilities (Zhang et al., 2007). Recently, Oliveira et al. (2022) successfully applied ANN models to predict egg-production traits in commercial laying breeder hens, demonstrating their high accuracy and effectiveness in modeling complex biological processes. Bishop (2006) emphasized that the inherent parallel and simultaneous processing capabilities of neural networks significantly improve computational efficiency, leading to their widespread use in solving complex problems across various fields. Multi-layer neural networks, as advanced nonlinear models, can simulate intricate relationships between inputs and outputs by utilizing nonlinear activation functions and adjusting weights across different layers. These networks are highly effective in identifying complex patterns, improving predictions, and supporting decision-making processes. The flexibility and adaptability of these networks make them highly suitable for addressing complex nonlinear challenges, control issues, and predicting critical economic factors such as egg production (Oliveira et al., 2022), growth and reproductive performance (Mehri, 2013), and nutritional requirements (Kaewtapee et al., 2021; Mehri, 2014).

This study provides a novel contribution by simultaneously evaluating the predictive performance of both classical nonlinear models and an ANN across multiple traits and commercial egg-type strains. Unlike previous studies that typically focus on a single trait or genetic line, our comparative approach integrates information across diverse breeder strains and production-related traits, offering a more comprehensive assessment of model efficiency and biological interpretation. This integrated framework enhances our understanding of model performance across genetically diverse populations and supports more precise decision-making in both nutrition and breeding programs.

## Materials and methods

### Data source

The dataset used in this study was compiled from published information on the objective performance of three widely used commercial broiler parent stocks (Ross 308, Cobb, and Arbor Acres) (Aviagen.com, 2021a, Aviagen.com, 2021b; Cobb-Vantress.com, 2025). For each strain, performance records were obtained from breeder management databases covering the hens' production phase for 40 consecutive weeks, beginning at 23 weeks for Ross 308, 24 weeks for Cobb, and 25 weeks for Arbor Acres, and ending at 62, 63, and 64 weeks of age for each strain, respectively. All data were manually entered into Excel spreadsheets and verified for accuracy and consistency before analysis. The traditional models and ANN were fitted to the data using the nonlinear regression procedure of SigmaPlot (SPSS, 1998) and the ANN option of GMP_pro_ software*.* This study used only previously published datasets and commercial management records with no new animal experimentation. All original data were collected under ethical standards established in the respective primary sources; therefore, additional institutional animal care or ethics approval was not required for the present secondary analysis.

### Modelling weekly egg production metrics

This analysis undertakes a comparative evaluation of three traditional egg production models, Gamma, Compartmental, and the Logistic-curvilinear models, in predicting weekly egg production metrics. Each model presents unique methodologies and assumptions regarding the dynamics of egg production, making it essential to assess their effectiveness in representing key production metrics. The metrics included in this study were: weekly egg production (%), reflecting the percentage of hens laying eggs within a given week; weekly egg mass (g), quantifying the total weight of eggs laid per hen over a week; weekly hatching egg (%), indicating the proportion of eggs suitable for incubation; and weekly egg weight (g), representing the average weight of individual eggs. The structure and properties of the models are as follows:

$$\text{Gamma}\;y=ax^{b}e^{-cx}$$

In the gamma model, y is the interested egg production metric, *a* is the initial production, *b* is the rate of increase to the peak, *c* is the rate of decrease after the peak, and *x* is the number of weeks of egg production. Additionally, the week of peak production (*b*/*c*) and persistency of peak production (–(*b* 1)×ln*c*) can be derived from the model parameters.

$$\text{Compartmental}\;y=a\;\left(e^{-\mathrm{bx}}-\;e^{-\mathrm{cx}}\right)$$

In the case of the compartmental model, y is the interested egg production metrics, *a* is the asymptotic value of egg production at the peak of egg-laying, *b* is the rate of production decrease after the peak (eggs/hen day decrease per week), *c* is the intermediate rate of weekly increase in egg-laying, and *x* is the number of weeks of egg production.

$$\text{Logistic}-\text{curvilinear}\;y=a\left(\frac{e^{-bx}}{1+e^{-c\left(x-d\right)}}\right)$$

In which, the biological interpretations of the relevant parameters for Logistic-curvilinear are: *y* = the interested egg production metrics, *a* = *a* scale parameter, *b* = the rate of decrease in laying ability, *c* = the reciprocal indicator of the variation in sexual maturity, *d* = the mean age of sexual maturity of the hens, and *x* is weeks of egg production.

### Modelling cumulative egg production

In the study of avian reproductive performance, cumulative egg production traits are pivotal metrics. These traits include: cumulative egg production (%), cumulative egg mass (kg), cumulative hatching egg (%), and cumulative egg weight (kg). Understanding these parameters is crucial for optimizing breeding strategies and improving overall productivity in poultry farming. To analyze the relationships between the aforementioned cumulative egg production metrics and week of egg production, five nonlinear models were employed. Below are the models used in this analysis:

$$\text{Gamma}\;y=ax^{b}e^{-cx}$$

$$\text{Logistic}-\text{curvilinear}\;y=a\left(\frac{e^{-bx}}{1+e^{-c\left(x-d\right)}}\right)$$

$$\text{Gompertz}\;y=b\;\text{exp}\;\left[\left(\text{ln}\;\frac{a}{b}\right)\left(1-e^{-cx}\right)\right]$$

$$\text{Richards}\;y=\;\frac{ab}{{\left[a^{n}+\left(a^{n\;}b^{n}\right)e^{-cx}\right]}^{1/n}}$$

$$\text{Morgan}\;w=\left(bk^{c}\;+\;a\;x^{c}\right)/\left(k^{c}+x^{c}\right)$$

In which the biological interpretations of the relevant parameters for Gamma, Compartmental, and the Logistic-curvilinear models are as noted earlier. For Gompertz, Richards, and Morgan: *y* = the interested egg production metric, *b* = initial value of cumulative egg production metrics, *a* = asymptotic value of cumulative egg production metric; *x* = weeks of egg production, *c, k,* and *n* are parameters that define the shape of the egg production curve.

### Artificial neural networks (ANN)

In addition to the traditional models, a multilayer perceptron (MLP) feed-forward ANN was developed to predict egg production performance. The ANN was used both for weekly and cumulative production data. Its architecture consisted of four layers: one input layer, two hidden layers, and one output layer. The ANN structure was configured with one input neuron, three neurons in the first hidden layer, two in the second hidden layer, and one output neuron (1-3-2-1). A hyperbolic tangent activation function was employed in both the hidden and output layers. Additionally, 33% of the dataset was allocated for validation to enhance the model's reliability. The ANN was trained to predict the egg production traits. After ANN training and the selection of the most adjusted network model for each trait of interest, the ANN models’ efficacy was tested. The predicted values for training and validation data sets were compared with the actual observed data to assess the ANN performance. The ANN was trained using JMP Pro software, which automatically allocated 33% of the dataset for validation to prevent overfitting. Because the model used a single biological input variable and a low-complexity architecture (1–3–2–1), no additional cross-validation cycles or regularization techniques were required.

### Statistical criteria

Various statistical criteria were used to assess the accuracy and reliability of both the traditional growth models and the ANN models. These criteria included the adjusted coefficient of determination (*R^2^*_adj_), root mean square error (RMSE), as well as model selection criteria such as Akaike’s information criterion (AIC) and Bayesian information criterion (BIC). The calculations for these statistical measures were performed as follows:

1) *R^2^*_adj_=1-[(*n*-1/*n-p*) (1-*R^2^*)]

where: *R^2^*_adj_ represents the adjusted coefficient of determination, *n* is the total number of observations, *p* denotes the number of parameters in the model, and *R^2^* is the standard coefficient of determination. The *R^2^* value itself is derived from the ratio of the residual sum of squares **(RSS)** to the total sum of squares **(TSS)**, providing a measure of how well the model explains the variability in the observed data.

1) RMSE=√(RSS/(n-p-1))

Where RSS is the residual sum of squares, n is the number of observations, and p is the number of parameters in the model.

2) MAD = ∑ (Ai-Fi)/n
3) MAPE =1/n ∑ | (Ai-Fi)/Ai | ×100
4) AIC = *n* × ln (RSS) + 2P
5) BIC = *n* ln (RSS/n) + *p* ln(n)

Where *A_i_* is the actual value, *F_i_* is the forecasted value, RSS is the residual sum of squares, *n* is the number of observations, and *p* is the number of parameters in the models. Except for *R^2^*_adj_, where higher values indicate a better fit of the model to the data, for the remaining studied criteria, the best model is the one with the lowest values**.**

## Results

A summary of the performance of the models based on statistically significant parameters (*P* < 0.05) estimated by each model in terms of percentage of the total fits is demonstrated in Table 1.

**Table 1:** A summary of the performance of the models based on statistically significant parameters (*P* < 0.05) estimated by each model in terms of the percentage of the total fits**.**

		Parameter					
Weekly egg production	Model	a	b	c	d	k	n
	Gamma	100	100	100	-	-	-
	Compartmental	100	100	100	-	-	-
	Logistic-curvilinear	100	100	100	100	-	-
Cumulative egg production	Gamma	100	100	100	-	-	-
	Compartmental	0	0	0	-	-	-
	Logistic-curvilinear	100	100	100	100	-	-
	Gompertz	100	100	100	-	-	-
	Richards	33.3	83.3	83.3	-	-	100
	Morgan	83.3	100	83.3	-	100	-

The results for the percentage of models' superiority based on AIC and BIC (Table 2, Table 3) demonstrated that the ANN provided the highest accuracy for weekly data across all strains and traits, which indicates the ANN's capacity to capture complex patterns within the data enables it to outperform the other models according to the goodness of fit criteria. When examining cumulative data, the Morgan model is shown as the most effective among classical growth models. This suggests a potential ability of the classical model, especially Morgan, in scenarios where accumulated effects over time are more relevant than immediate fluctuations. Although other classical models’ efficacy, such as Gamma, Logistic-curvilinear, and Richards, were acceptable in most cases, they were less consistent than the ANN and Morgan.

**Table 2:** Comparison of goodness of fit showing the efficacy of egg production curves and ANN to predict egg production traits in Ross 308, Cobb, and Arbor Acres parent stocks.

			Ross 308				Cobb				Arbor Acres			
Traits	Model	Parameter	*R*^2^ adj	RMSE 1	AIC 2	BIC 3	*R*^2^ adj	RMSE 1	AIC 2	BIC 3	*R*^2^ adj	RMSE 1	AIC 2	BIC 3
Egg Mass (kg)	Gamma	Weekly	0.8393	0.0049	−134.39	−276.88	0.8679	0.0044	−145.23	−297.00	0.8286	0.0052	−132.56	−275.05
		Cumulative	0.9996	0.2013	−58.118	−200.60	0.9997	0.0250	−72.41	−224.17	0.9997	0.0291	−63.81	−206.29
	Compartmental	Weekly	0.9169	0.0025	−160.93	−303.42	0.9165	0.0028	−164.39	−316.16	0.9060	0.0028	−154.51	−299.00
		Cumulative	0.9942	3.2558	−53.217	−89.271	0.9933	0.5568	57.79	−93.97	0.9948	0.4493	45.67	−96.81
	Logistic Curvilinear	Weekly	0.9809	0.0005	−218.19	−358.99	0.9576	0.0013	−192.24	−342.27	0.9665	0.0010	−196.64	−337.43
		Cumulative	0.9981	1.0462	9.806	−130.99	0.9979	0.1718	9.86	−138.11	0.9980	0.1734	9.03	−131.76
	Gompertz	Cumulative	0.9966	1.9130	−31.94	−110.54	0.9969	0.2575	25.41	−126.35	0.9965	0.3066	30.39	−112.09
	Richard	Cumulative	0.9996	0.2046	−55.46	−196.26	0.9997	0.0237	−73.33	−223.36	0.9996	0.0303	−60.74	−201.54
	Morgan	Cumulative	0.9999	0.0444	−116.58	−257.38	0.9999	0.0056	−133.28	−281.26	0.9999	0.0065	−121.97	−262.77
	ANN (training)	Weekly	0.9998	0.0010	−252.68	−324.81	0.9999	0.0003	−338.32	−418.30	0.9912	0.0004	−292.45	−364.58
		Cumulative	0.9999	0.0009	−180.44	−265.15	0.9999	0.0288	−90.86	−174.75	0.9999	0.0002	−212.12	−296.83
	ANN (Validation)	Weekly	0.9990	0.0008	−140.66	−171.22	0.9996	0.0012	−129.43	−159.98	0.9915	0.0013	−128.70	−159.26
		Cumulative	0.9999	0.0004	−108.18	−145.13	0.9998	0.0436	−43.10	−79.81	0.9999	0.0003	−109.75	−146.69
Egg production (Eggs/birdk)	Gamma	Weekly	0.8022	1.7226	99.42	−43.06	0.8270	1.6606	103.69	−48.07	0.7892	1.8457	102.18	−40.30
		Cumulative	0.9995	10.1685	170.44	27.95	0.9995	10.1310	179.64	27.87	0.9995	10.9746	173.49	31.00
	Compartmental	Weekly	0.8972	0.8953	73.24	−69.24	0.8985	0.9737	81.27	−70.49	0.8958	0.9123	73.99	−68.48
		Cumulative	0.9943	120.0242	267.17	126.68	0.9938	125.8439	285.46	133.69	0.9949	11.5799	266.61	124.12
	Logistic Curvilinear	Weekly	0.9927	0.0631	−31.39	−172.19	0.9841	0.1507	4.36	−145.66	0.9944	0.0483	−42.08	−182.88
		Cumulative	0.9970	48.8881	234.68	93.88	0.9974	46.4799	245.06	95.03	0.9977	51.3181	236.62	95.82
	Gompertz	Cumulative	0.9960	85.5029	255.61	113.12	0.9960	82.3034	267.62	115.85	0.9958	93.4855	259.18	116.69
	Richard	Cumulative	0.9995	9.7671	170.27	29.47	0.9995	9.1009	176.58	26.54	0.9995	10.9421	174.81	34.01
	Morgan	Cumulative	0.9999	2.1360	109.46	−31.33	0.9999	1.8803	110.35	−39.68	0.9999	2.2442	111.44	−29.35
	ANN (training)	Weekly	0.9999	0.0571	−82.49	−155.28	0.9992	0.0382	−71.78	−153.29	0.9996	0.0243	−89.20	−161.84
		Cumulative	0.9999	0.0533	−39.03	−69.72	0.9999	0.0356	−14.27	−45.78	0.9999	0.0533	−62.58	−136.20
	ANN (Validation)	Weekly	0.9997	0.1479	−88.10	−160.76	0.9965	0.0458	−30.17	−60.99	0.9983	0.0368	−35.97	−66.70
		Cumulative	0.9999	0.1015	−43.63	−74.29	0.9999	0.0874	−75.45	−156.76	0.9999	0.1015	−48.66	−79.28
Hatching egg (%)	Gamma	Weekly	0.9919	2.3470	111.68	−30.80	0.9850	1.6210	102.67	−49.09	0.9975	0.4015	41.17	−101.31
		Cumulative	0.9994	11.7403	176.19	33.70	0.9994	10.8915	182.68	30.91	0.9994	11.7214	176.12	33.63
	Compartmental	Weekly	0.8158	53.0031	236.48	93.99	0.4912	55.0108	250.70	98.93	0.7854	34.4327	219.23	−76.74
		Cumulative	0.9921	144.31	276.55	134.06	0.9926	127.2044	285.91	134.14	0.9934	120.0300	269.18	126.69
	Logistic Curvilinear	Weekly	0.9809	5.4259	146.75	5.95	0.9948	0.5522	58.89	−91.13	0.9963	0.5911	58.07	82.72
		Cumulative	0.9978	39.6647	226.32	85.52	0.9974	43.5697	242.35	92.32	0.9978	39.8097	226.47	85.67
	Gompertz	Cumulative	09962	69.44	247.29	104.80	0.9959	430.8478	260.76	108.99	0.9960	73.5391	249.58	107.09
	Richard	Cumulative	0.9994	10.5974	173.53	32.73	0.9994	9.6940	179.23	29.20	0.9994	11.0310	175.13	34.33
	Morgan	Cumulative	0.9999	0.8546	72.82	−67.97	0.9999	2.4061	120.70	−29.32	0.9999	0.6992	64.79	−76.00
	ANN (training)	Weekly	0.9998	0.0907	−31.21	−110.34	0.9996	0.0727	−42.71	−115.37	0.9998	0.0721	−39.05	−115.61
		Cumulative	0.9999	0.0347	−71.66	−144.82	0.9999	0.0247	−97.34	−177.86	0.9999	0.0814	−34.64	−112.42
	ANN(Validation)	Weekly	0.9999	0.0999	−24.99	−52.93	0.9995	0.0987	−11.70	−43.48	0.9999	0.0262	−41.53	−69.29
		Cumulative	0.9999	0.0456	−27.63	−55.52	0.9999	0.0848	−14.95	−46.41	0.9999	0.0658	−18.86	−46.97
Egg weight	Gamma	Weekly	0.9970	0.6342	59.45	−83.03	0.9936	1.5503	100.80	−50.96	0.9970	0.5447	53.37	−89.11
		Cumulative	0.9999	0.000004	−411.61	−554.10	0.9999	0.00001	−380.83	−532.60	0.9999	0.00001	−381.17	−523.66
	Compartmental	Weekly	0.9293	14.8293	185.53	43.04	0.9012	23.8893	215.67	63.90	0.9459	9.7517	168.76	26.27
		Cumulative	0.9999	0.0005	−225.05	−367.54	0.9999	0.0003	−257.168	−408.936	0.9999	0.0003	−244.63	−387.12
	Logistic Curvilinear	Weekly	0.9995	0.0942	−15.38	−156.18	0.9994	0.1467	3.218	−146.81	0.9977	0.4006	42.52	−98.27
		Cumulative	0.9991	0.0030	−152.47	−293.27	0.9991	0.0036	−152.30	−300.28	0.9991	0.0030	−152.47	−293.27
	Gompertz	Cumulative	0.9975	0.0086	−112.41	−254.90	0.9977	0.0094	−113.51	−265.28	0.9976	0.0082	−114.23	−256.72
	Richard	Cumulative	0.9999	0.00005	−316.46	−457.26	0.9999	0.00003	−349.72	−499.75	0.9999	0.00005	−316.46	−457.26
	Morgan	Cumulative	0.9999	0.000008	−387.11	−527.91	0.9999	0.000001	−493.78	−641.76	0.9999	0.00001	−360.41	−501.21
	ANN (training)	Weekly	0.9999	0.0465	−57.75	−131.72	0.9999	0.0275	−89.11	−169.88	0.9999	0.0293	−80.01	−152.87
		Cumulative	0.9999	0.00003	−267.82	−352.53	0.9999	0.00004	−262.26	−346.97	0.9999	0.00004	−263.27	−347.98
	ANN (Validation)	Weekly	0.9999	0.0383	−47.33	−77.96	0.9999	0.0252	−46.31	−76.95	0.9999	0.0317	−40.00	−70.68
		Cumulative	0.9999	0.00003	−144.58	−181.53	0.9999	0.00007	−133.37	−170.32	0.9999	0.00000004	−237.45	−274.25

1 Root Mean squared error.

2 Akaike’s information criterion.

3 Bayesian information criterion.

**Table 3:** Percentage of cases in which the model specified in the row was superior to the model specified in the column based on Akaike’s information criterion (AIC) and Bayesian information criterion (BIC).

	Parameter							
	Model	Gama		Compartmental	Logistic-curvilinear			
Weekly egg metrics	Gama	AIC	-	50	16.7			
		BIC	-	50	16.7			
	Compartmental	AIC	50	-	0			
		BIC	50	-	0			
	Logistic-curvilinear	AIC	83.3	100	-			
		BIC	83.3	100	-			
	ANN(training)	AIC	100	100	91.6			
		BIC	100	100	58.3			
	ANN(validation)	AIC	83.3	75	66.6			
		BIC	33.3	50	16.6			
Cumulative egg metrics			Gama	Compartmental	Logistic-curvilinear	Gompertz	Richards	Morgan
	Gama	AIC	-	100	100	100	50	16.6
		BIC	-	100	100	100	75	16.6
	Compartmental	AIC	0	-	33.3	33.3	0	0
		BIC	0	-	25	25	0	0
	Logistic-curvilinear	AIC	0	64.7	-	91.6	0	0
		BIC	0	75	-	100	0	0
	Gompertz	AIC	50	36.7	9.4	-	0	0
		BIC	25	75	0	-	0	0
	Richards	AIC	50	100	100	100	-	0
		BIC	25	100	100	100	-	0
	Morgan	AIC	83.4	100	100	100	100	-
		BIC	83.4	100	100	100	100	-
	ANN(training)	AIC	75	100	100	100	75	66.6
		BIC	66.6	75	100	100	66.6	66.6
	ANN(validation)	AIC	66.6	75	83.3	100	66.6	50
		BIC	50	66.6	75	75	50	33.3

Percentage of cases in which the model specified in the row was statistically superior to the model specified in the column.

**Fig. 1:**
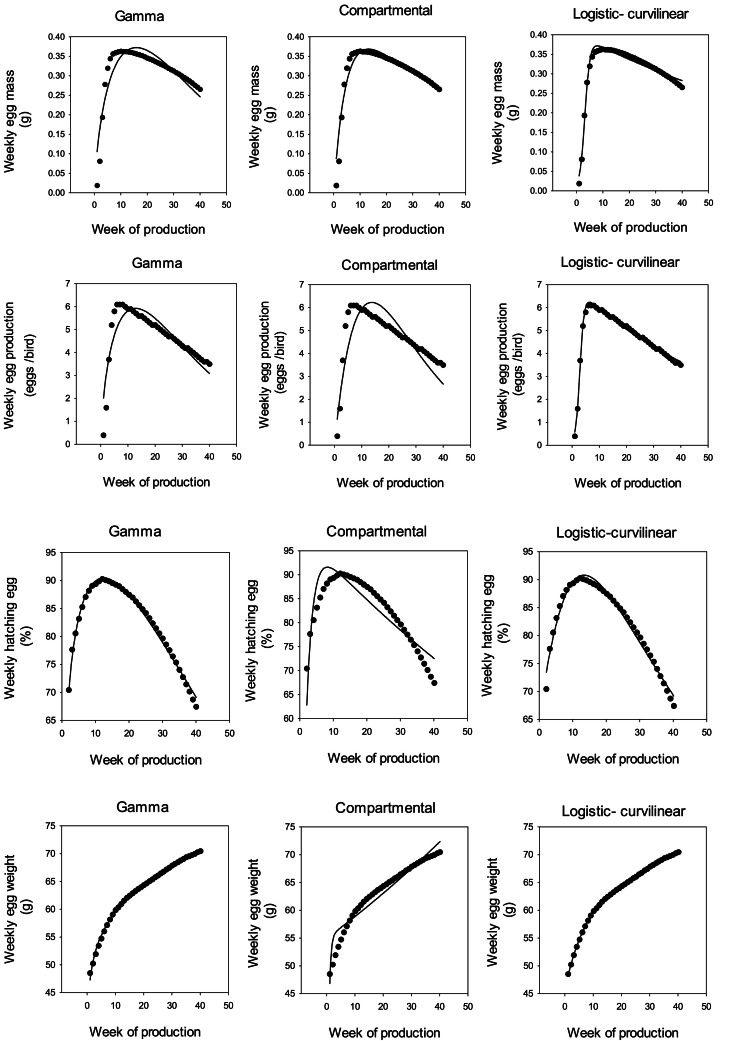
Weekly egg mass (g), egg production (eggs/ bird /week), hatching egg (%), and egg weight (g) curves of Ross 308 parent stock.

**Fig. 2:**
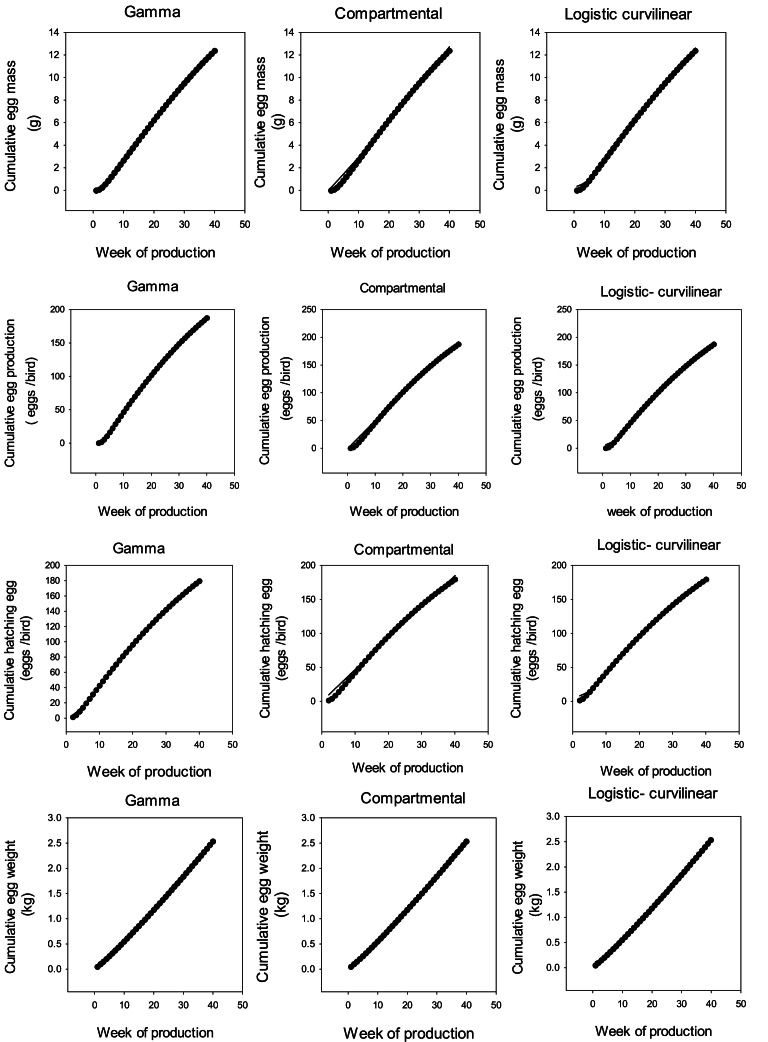
Cumulative egg mass (kg), egg production (eggs/bird), hatching egg (ess/bird), and egg weight curves (kg) of Ross 308 parent stock.

**Fig. 3:**
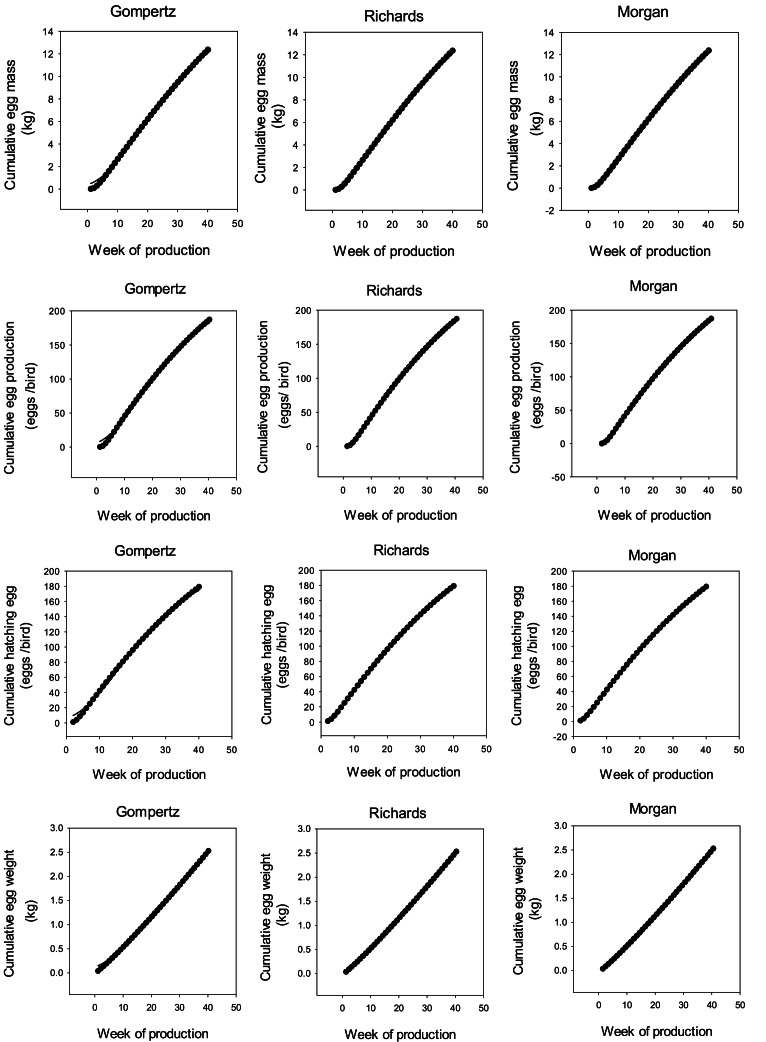
Cumulative egg mass (kg), egg production (eggs/bird), hatching egg (eggs/bird), and egg weight (kg) curves of Ross 308 parent stocks.

Limitations on space prevent the presentation of the results for all data sets. Therefore, as an example, model behavior for Ross 308 parent stock data set is illustrated in Figs. 1 and 7. The results indicate that the growth functions and ANN could be fitted to the data without difficulty by nonlinear regression. Although in some cases, the compartmental model was not able to show a suitable fit to the data, the other models provided an excellent fit. Poorer fits of the Gamma and Logistic-curvilinear occurred less with the cumulative data than the weekly data, which is in line with the results obtained for the ANN (Table 1, Table 2, Table 3, Fig. 2, Fig. 3).

**Fig. 4:**
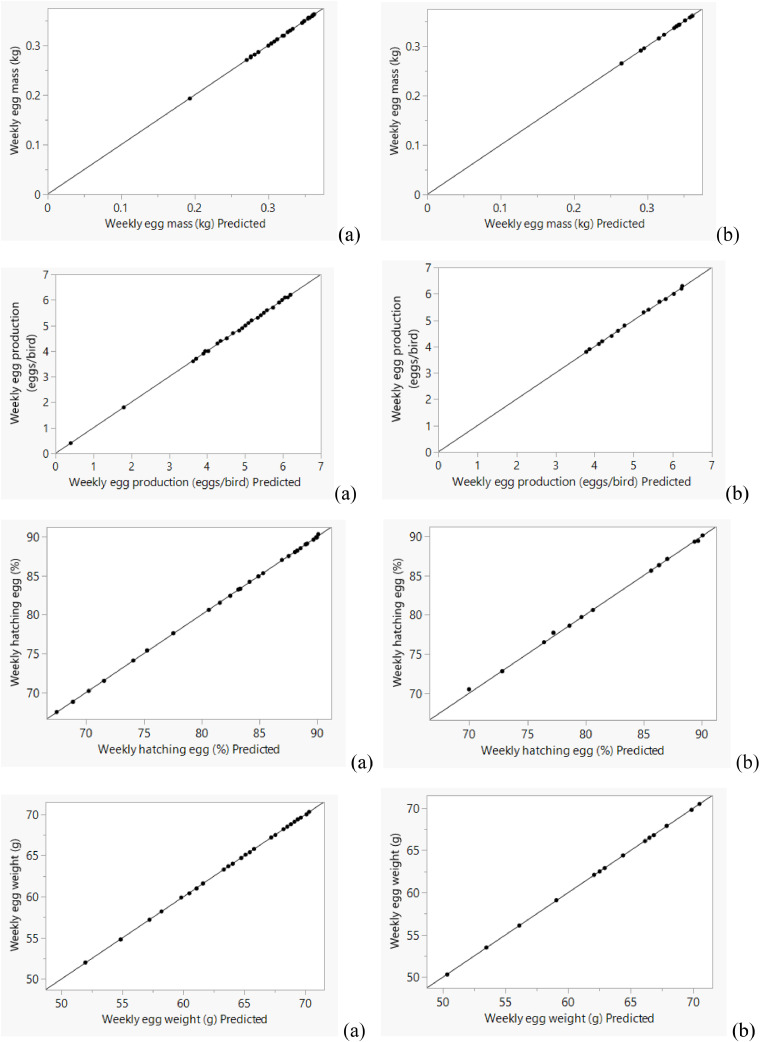
Actual *vs.* predicted plot by artificial neural network for weekly training (a) and validating (b) data sets of Ross 308 parent stocks.

**Fig. 5:**
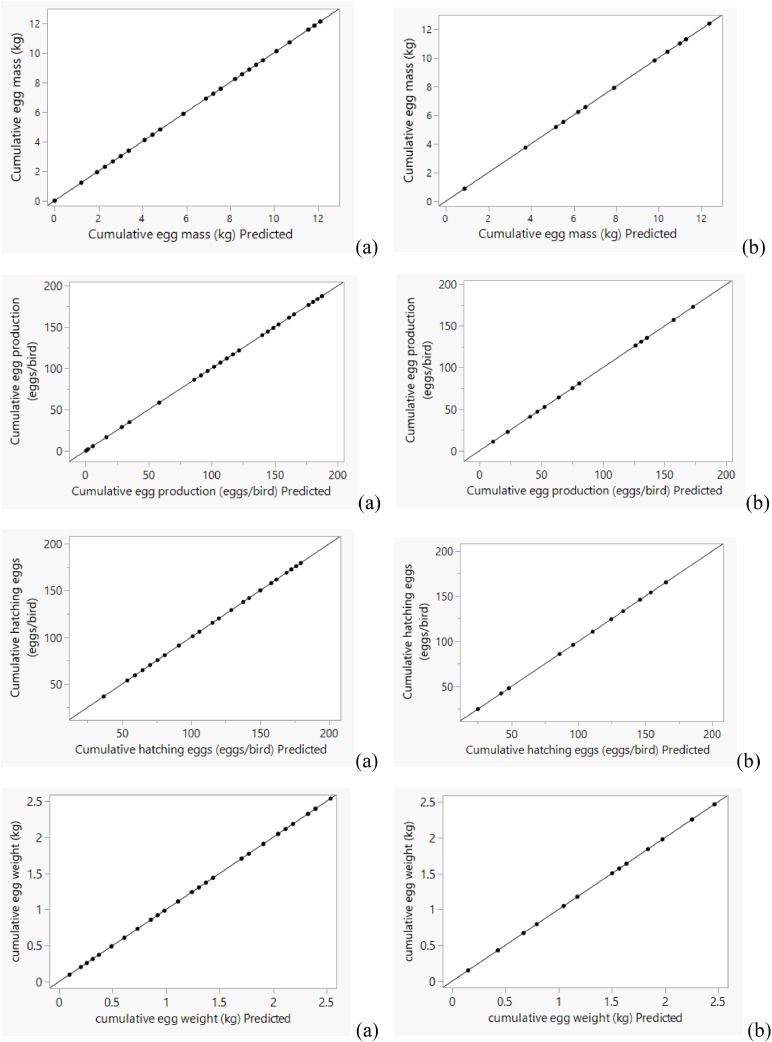
Actual *vs.* predicted plot by artificial neural network for cumulative training (a) and validating (b) data sets of Ross 308 parent stocks.

**Fig. 6:**
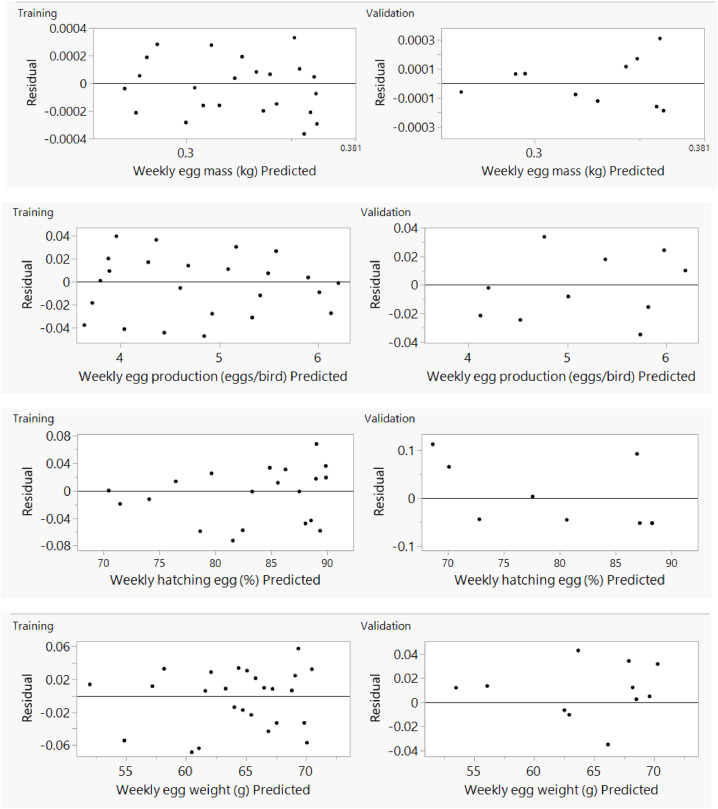
Residual *vs*. predicted plot by artificial neural network for weekly training and validating data sets of Ross 308 parent stocks.

**Fig. 7:**
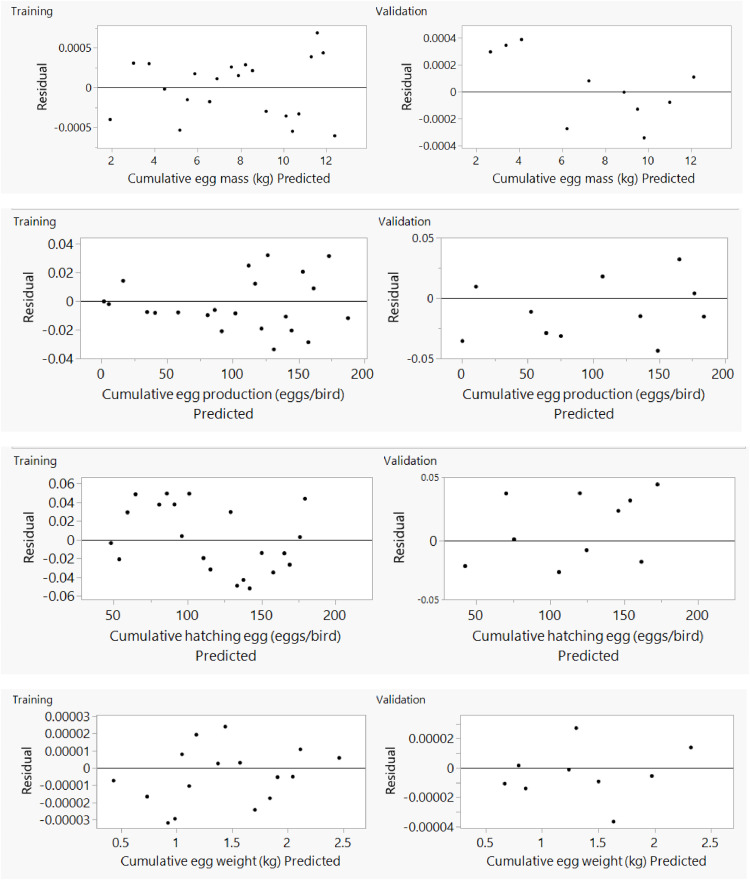
Residual *vs*. predicted plot by artificial neural network for cumulative training and validating data sets of Ross 308 parent stocks.

The ANN accurately captured the actual variations in both weekly and cumulative data (Fig. 4, Fig. 5, Fig. 6, Fig. 7), with predictions closely matching the observed values.

The higher percentages of fitted cases (Table 2, Table 3) in which the ANN was superior to the best traditional Logistic-curvilinear and Morgan models according to the RMSE, AIC and BIC criteria (RMSE = 100% both for weekly and cumulative productive metrics, AIC = 91.6% for weekly and 58.3% for cumulative datasets, and BIC = 66.6% both for weekly and cumulative datasets), represent the capacity of the ANN to outperform the conventional models. The AIC and BIC are crucial statistical measures used to evaluate the quality of different models while penalizing for complexity. A lower AIC or BIC value indicates a better-fitting model. Thus, the higher percentages observed for the ANN suggest a robust explanatory capability that surpasses its traditional counterparts due to its better ability to represent the real physiological and reproductive responses of breeder hens to feeding level, age, and strain differences. From a management perspective, the improved accuracy means that flock reproductive performance can be predicted more precisely, allowing earlier detection of production drops or efficiency losses. Such predictive ability enables producers to adjust feeding programs, lighting schedules, or strain selection in time, leading to better resource use and improved overall reproductive productivity in broiler parent stocks.

## Discussion

The poultry industry faces continuous pressure to improve production efficiency through advanced management techniques. Accurate prediction of egg production parameters plays a pivotal role in sustainable flock management. Classical growth models have long been employed to forecast production trends; however, recent advancements in machine learning, particularly ANN, offer promising alternatives that may enhance predictive capabilities (Roush et al., 2006; Khojastehkey et al., 2021). Accurate prediction of key production parameters, such as egg mass, hatching egg production, egg weight, and overall egg production throughout the production cycle, plays a crucial role in optimizing flock management and enhancing productivity in the poultry industry.

This study explores the performance of classical growth models and ANNs in predicting weekly and cumulative production patterns across three commercial parent stocks of broiler chicks. Gamma, Compartmental, and Logistic curvilinear models were applied to analyze the weekly data, while Gamma, Compartmental, Logistic curvilinear, Gompertz, Richards, and Morgan models were used for the cumulative data. A multi-layer feed-forward perceptron neural network structure was also employed for the ANN model. Model performance was assessed using *R²*_adj_, RMSE, AIC, and BIC criteria.

The outcomes of the current study align with those reported by Darmani Kuhi and France (2019), who successfully applied classical growth functions to model cumulative egg production in laying and breeder hens. Traditional analysis methods often exhibit sensitivity to missing records, which can lead to skewed results and misinterpretations in production trends. In this regard, the application of non-linear growth functions presents a compelling alternative for analyzing cumulative egg yield records. Unlike conventional models, these non-linear approaches demonstrate an inherent resilience to missing data points. This characteristic is particularly advantageous in agricultural settings where data collection may be sporadic or incomplete due to various factors such as environmental changes or operational challenges. The smoother trend depicted by cumulative production curves fitted with non-linear functions offers a more robust representation of egg yield over time and is less influenced by outliers or abnormal records. Abnormal records can be a result of several problems, such as measurement faults and biological disruptions in animal performance occurring when birds are threatened by some severe condition that limits expression of their genetic potential, e.g., nutritional deficit, metabolic or infectious disorders (Jefferson, 2005).

Despite the fact that flexible growth functions do not always produce statistically significant parameter estimates (Table 3), it is crucial to recognize that this aspect should not be the only determining factor in the selection process for a model (Darmani Kuhi and France, 2019). Considering the Compartmental model, for example, even when the statistical criteria did not indicate a suitable fit across the different data sets, fitting the Compartmental model consistently resulted in statistically significant parameter estimates.

The superior performance of the ANN model is likely due to its ability to capture complex and nonlinear interactions among nutrition, genetic potential, and reproductive physiology in broiler breeder hens. These relationships often involve multifactorial effects between nutritional and physiological factors; for example, the combination of feed level, body weight, and age can nonlinearly influence egg production and fertility, as also reported by Van Emous et al. (2021). From a management perspective, the outputs of the ANN can serve as an effective decision-support tool for flock management. Accurate prediction of weekly and cumulative egg production trends allows early detection of performance declines, enabling timely adjustments to feeding programs, lighting schedules, or breeder management strategies. Moreover, these predictions can assist in optimizing resource utilization, improving feed efficiency, and reducing production costs under different nutritional or environmental conditions.

Although the ANN model demonstrated high predictive accuracy in predicting production traits of broiler parent stocks, certain limitations should be acknowledged. The model was developed using data from a specific set of breeder strains and management conditions, which may limit its generalizability to other genetic lines or production systems. Furthermore, it relied primarily on historical production and physiological data without incorporating real-time environmental or behavioral inputs. Future research should focus on integrating ANN models with emerging technologies such as the Internet of Things (IoT) and real-time monitoring systems to collect continuous data on environmental parameters, bird behavior, and feeding activity. Such integration could enhance predictive precision and support more dynamic and intelligent management decisions for broiler parent flocks (Cruz et al., 2024).

Overall, the findings of this study confirm the high potential of ANNs as powerful tools for modeling and predicting egg production parameters in laying hens, while also acknowledging the applicability of classical models in predicting the egg production metrics.

## Conclusion

Based on the comparative analysis of various growth models and ANNs for predicting productive parameters across three commercial broiler breeder strains, ANNs consistently demonstrated the highest accuracy and the lowest prediction error, especially in weekly data modeling. In the analysis of production metrics, the ANN demonstrated a significant advantage over the best traditional Logistic curvilinear and Morgan models based on RMSE, AIC, and BIC criteria (RMSE = 100% both for weekly and cumulative productive metrics, AIC = 91.6% for weekly and 58.3% for cumulative datasets, and BIC = 66.6% both for weekly and cumulative datasets. Classical models such as Morgan and Richard performed well in modeling cumulative data, sometimes approaching the predictive accuracy of ANN. Overall, it can be concluded that both approaches, classical growth functions and neural networks, can serve as complementary and effective tools in modeling egg production, depending on the type of data and analytical objectives. While classical models, due to their simplicity and interpretability, are more suitable for quick and transparent analyses, neural networks offer superior predictive accuracy and the capability to model complex, nonlinear relationships. Integrating both methods could provide a powerful strategy for optimizing flock performance and enhancing productivity in poultry production systems by implementing them in breeding programs or management software.

## Ethics approval statement

This study did not involve direct experimentation on animals, and therefore, ethics approval was not required.

## CRediT authorship contribution statement

**Zahra Moradi Gharajeh:** Methodology, Formal analysis, Data curation. **Hassan Darmani Kuhi:** Writing – original draft, Methodology, Formal analysis, Conceptualization. **Navid Ghavi Hossein-Zadeh:** Writing – review & editing, Formal analysis.

## Disclosures

There is no Conflict of Interest

## Appendix Supplementary materials

Image, application 1
