# Supplementary material for: Comparative analysis of classical growth models and artificial neural networks in predicting egg production parameters in three commercial broiler parent stocks
Source: Poult Sci. 2025 Dec 28;105(3):106356. doi: 10.1016/j.psj.2025.106356 (PMC12828518; doi:10.1016/j.psj.2025.106356)
Supplement: Supplementary file 1 [file mmc1.docx]

**Supplementary Tables**

| **Table 1.** Estimated parameters for weekly egg production traits using Gamma, Compartmental, and Logistic-curvilinear models | | | | | | | | | | | | |
| --- | --- | --- | --- | --- | --- | --- | --- | --- | --- | --- | --- | --- |
|  | **Ross308** | | | | **Cobb** | | | | **Arbor Acres** | | | |
| **Model** | **Weekly EP** | **Weekly EM** | **Weekly EW** | **Weekly Hatchability** | **Weekly EP** | **Weekly EM** | **Weekly EW** | **Weekly Hatchability** | **Weekly EP** | **Weekly EM** | **Weekly EW** | **Weekly Hatchability** |
| **Gamma** | |  |  |  |  |  |  |  |  |  |  |  |
| a | 2.1274  ± 0.2557 | 0.1103  ± 0.0125 | 47.3324  ± 0.2063 | 61.3305 ±0.4987 | 1.963  ± 0.2404 | 0.0996  ± 0.0110 | 46.3587  ± 0.3118 | 71.3183  ± 0.3393 | 2.2634  ± 0.2699 | 0.1178  ± 0.0129 | 48.2689  ± 0.1932 | 71.5097  ± 0.2234 |
| b | 0.6616  ± 0.0725 | 0.6904  ± 0.0656 | 0.0937  ± 0.0027 | 0.2558  ±0.0050 | 0.6981  ± 0.0730 | 0.7525  ± 0.0630 | 0.1099  ± 0.0040 | 0.1414 ±0.0030 | 0.6355  ± 0.0721 | 0.6581  ± 0.0626 | 0.0758  ± 0.0025 | 0.1691  ± 0.0019 |
| c | 0.0516  ± 0.0049 | 0.0436  ± 0.0041 | -0.0014 ±0.0002 | 0.0206  ±0.0003 | 0.0560  ± 0.0049 | 0.0496  ± 0.0039 | -0.0005  ± 0.0003 | 0.0107  ± 0.0002 | 0.0498  ± 0.0049 | 0.0439  ± 0.0039 | -0.0022  ± 0.0002 | 0.0142  ± 0.0001 |
| **Compartmental** | |  |  |  |  |  |  |  |  |  |  |  |
| a | 8.1378  ± 0.4643 | 0.4770  ± 0.0257 | 55.0911  ± 0.5008 | 99.0852  ± 1.6050 | 8.3353 ± 0.5440 | 0.5255 ± 0.0355 | 55.6265 ± 0.6114 | 89.4481  ± 1.1313 | 8.1147  ± 0.4306 | -0.4747  ± 0.0241 | 54.5156  ± 0.4040 | 96.8920  ± 1.1048 |
| b | 0.0214  ± 0.0022 | 0.0144  ± 0.0019 | -0.0068  ± 0.0004 | 0.0078  ± 0.0007 | 0.0253  ± 0.0024 | 0.0201  ± 0.0022 | -0.0067  ± 0.0004 | 0.0022  ± 0.0005 | 0.0204  ± 0.0020 | 0.2342  ± 0.0213 | -0.0066  ± 0.0003 | 0.0053  ± 0.0005 |
| c | 0.2605  ± 0.0262 | 0.2211  ± 0.0203 | 1.8667  ± 0.5008 | 0.5258  ± 0.0342 | 0.2432  ± 0.0256 | 0.1926  ± 0.0184 | 1.7473  ± 0.2006 | 1.3904  ± 0.1315 | 0.2739  ± 0.0272 | 0.0163  ± 0.0018 | 2.1059  ± 0.1949 | 0.7260  ± 0.0472 |
| **Logistic -curvilinear** | | |  |  |  |  |  |  |  |  |  |  |
| a | 7.0278  ± 0.0564 | 0.4021  ± 0.0051 | 59.4610  ± 0.1377 | 117.7231  ± 2.4673 | 6.9690  ± 0.0871 | 0.4129  ± 0.0082 | 62.7389  ± 0.2526 | 99.8977  ± 0.3962 | 7.1209  ± 0.0484 | 0.4048  ± 0.0064 | 60.1034  ± 0.5670 | 109.0112  ± 0.6567 |
| b | 0.0164  ± 0.0004 | 0.0088  ± 0.0005 | -0.0044  ±0.00007 | 0.0132  ±0.0006 | 0.0193  ±0.0006 | 0.0125  ± 0.0008 | -0.0031  ± 0.0001 | 0.0059  ± 0.0001 | 0.0159  ± 0.0003 | 0.0112  ± 0.0007 | -0.0036  ± 0.0003 | 0.0091  ± 0.0002 |
| c | 1.2356  ± 0.0499 | 1.0169  ± 0.0628 | 0.1947  ± 0.0047 | 0.1646  ±0.0112 | 1.2786  ± 0.0808 | 0.9734  ± 0.0891 | 0.1469  ± 0.0038 | 0.2086  ± 0.0056 | 1.2663  ± 0.0446 | 1.0837  ± 0.0877 | 0.1311  ± 0.0096 | 0.1703  ± 0.0056 |
| d | 2.9171  ± 0.0374 | 3.2003  ± 0.0687 | -6.5047  ± 0.1719 | -1.5314 ±0.2309 | 3.0027  ± 0.0569 | 3.3770  ± 0.1072 | -7.1648  ± 0.1713 | -3.8949  ± 0.1283 | 2.8311  ± 0.0317 | 3.0479  ± 0.0842 | -10.9588  ± 0.6430 | -4.3414  ± 0.1623 |
| 1. Weekly EP = weekly egg production; Weekly EM = weekly egg mass; Weekly EW = weekly egg weight; Weekly Hatchability = weekly percentage of hatching eggs. 2. a, b, c, d = model parameters estimated by nonlinear regression; values are presented as mean ± standard error (SE). | | | | | | | | | | | | |

| **Table 2.** Estimated parameters for cumulative egg production traits using Gamma, Compartmental, Logistic-curvilinear, Gompertz, Richards, and Morgan models | | | | | | | | | | | | |
| --- | --- | --- | --- | --- | --- | --- | --- | --- | --- | --- | --- | --- |
|  | **Ross308** | | | | **Cobb** | | | | **Arbor Acres** | | | |
| **Model** | **Cum.**  **EP** | **Cum.**  **EM** | **Cum.**  **EW** | **Cum. Hatchability** | **Cum.**  **EP** | **Cum.**  **EM** | **Cum.**  **EW** | **Cum. Hatchability** | **Cum.**  **EP** | **Cum.**  **EM** | **Cum.**  **EW** | **Cum. Hatchability** |
| **Gamma** | |  |  |  |  |  |  |  |  |  |  |  |
| a | 2.0275  ± 0.1095 | 0.0957  ± 0.0052 | 0.0454  ± 0.0001 | 1.5258 ±0.1000 | 1.9249  ± 0.1013 | 0.0871  ± 0.0043 | 0.0437  ± 0.0002 | 1.5905  ± 0.1015 | 2.2180  ± 0.1195 | 0.1084  ± 0.0054 | 0.0467  ± 0.0002 | 1.8257  ± 0.1137 |
| b | 1.4383  ± 0.0248 | 1.5134  ± 0.0246 | 1.0794  ± 0.0013 | 1.5296 ±0.0300 | 1.4461  ± 0.0238 | 1.5513  ± 0.0224 | 1.0967  ± 0.0022 | 1.5107  ± 0.0293 | 1.4109  ± 0.0248 | 1.4638  ± 0.0226 | 1.0628  ± 0.0019 | 1.4642  ± 0.0286 |
| c | 0.0197  ± 0.0010 | 0.0182  ± 0.0010 | -0.0010  ±5.3946E-05 | 0.0221 ±0.0012 | 0.0206  ± 0.0009 | 0.0205  ± 0.0009 | -0.0005  ±8.6910E-05 | 0.0228  ± 0.0012 | 0.0187  ± 0.0010 | 0.0174  ± 0.0009 | -0.0015  ±7.9283E-05 | 0.0204  ± 0.0012 |
| **Compartmental** | |  |  |  |  |  |  |  |  |  |  |  |
| a | 453.5960  ±3548.9870 | 75.3154  ±5685.5460 | 75.9773  ±26409.7425 | 465.4906  ± 8.8235 | 1.4345E+05±7.5143E+09 | 77.9332  ±6881.2704 | 77.0901±22440.8590 | 1284.0856  ±1.1129E+05 | 1.4877E+05±1.7726E+10 | 74.0530  ±4829.4956 | 77.0901  ±2.2441E+04 | -1265.0758  ±90275.4558 |
| b | -0.0038  ± 0.0402 | -0.0044  ± 0.1392 | -0.0046  ± 0.1223 | -0.0044 ±0.0537 | 0.0030  ± 3.3691 | -0.0036  ± 0.1554 | -0.0044  ± 0.1003 | -1.5292E-05± 0.1533 | 0.0017  ± 2.1185 | -0.0034  ± 0.1240 | -0.0044  ± 0.1003 | 0.0033  ± 0.1427 |
| c | 0.0075  ± 0.0492 | -0.0006  ± 0.1492 | -0.0038  ± 0.1239 | 0.0057 ±0.0645 | 0.0030  ± 3.3714 | 4.8227E-05±0.1659 | -0.0037  ± 0.1015 | 0.0036  ± 0.1637 | 0.0018  ± 2.1199 | 0.0006  ± 0.1332 | -0.0037  ± 0.1015 | -0.0006  ± 0.1332 |
| **Logistic -curvilinear** | | |  |  |  |  |  |  |  |  |  |  |
| a | 67.8769  ± 2.5525 | 3.9812  ± 0.1548 | 0.6922  ± 0.0213 | 64.8148 ±2.4822 | 65.9865  ± 2.5783 | 4.0281  ± 0.1680 | 0.7414  ± 0.0232 | 63.0787  ± 2.4640 | 69.1492  ± 2.5614 | 3.9773  ± 0.1546 | 0.6922  ± 0.0213 | 65.5971  ± 2.4371 |
| b | -0.0260  ± 0.0011 | -0.0289  ± 0.0011 | -0.0328  ± 0.0008 | -0.0260 ±0.0011 | -0.0251  ± 0.0011 | -0.0274  ± 0.0012 | -0.0311  ± 0.0008 | -0.0253  ± 0.0011 | -0.0262  ± 0.0011 | -0.0282  ± 0.0011 | -0.0328  ± 0.0008 | -0.0259  ± 0.0011 |
| c | 0.2396  ± 0.0132 | 0.2325  ± 0.0125 | 0.1961  ± 0.0088 | 0.2363 ±0.0127 | 0.2407  ± 0.0136 | 0.2285  ± 0.0124 | 0.1846  ± 0.0081 | 0.2446  ± 0.0138 | 0.2404  ± 0.0133 | 0.2312  ± 0.0126 | 0.1961  ± 0.0088 | 0.2362  ± 0.0127 |
| d | 10.1047  ± 0.3149 | 10.6758  ± 0.3236 | 9.0378  ± 0.2989 | 10.5639 ±0.3125 | 10.1710  ± 0.3244 | 10.9243  ± 0.3437 | 9.6856  ± 0.3178 | 10.3515  ± 0.3195 | 9.9535  ± 0.3124 | 10.4396  ± 0.3274 | 9.0378  ± 0.2989 | 10.2322  ± 0.3078 |
| **Gompertz** | |  |  |  |  |  |  |  |  |  |  |  |
| a | 213.5405± 4.8626 | 14.8659  ± 0.3853 | 3.7018  ± 0.1362 | 205.5755 ±4.6189 | 202.2969  ± 4.0445 | 13.8944  ± 0.3189 | 3.9282  ± 0.1361 | 189.8262  ± 4.0655 | 219.9989  ± 5.1687 | 14.3218  ± 0.3636 | 3.6655  ± 0.1349 | 206.9088  ± 4.7629 |
| b | 6.6785  ± 0.7866 | 0.3757  ± 0.0443 | 0.1324  ± 0.0093 | 5.7485 ±0.6990 | 6.5012  ± 0.7462 | 0.3447  ± 0.0396 | 0.1371  ± 0.0093 | 5.3843  ± 0.6814 | 7.1513  ± 0.8393 | 0.3989  ± 0.0454 | 0.1337  ± 0.0092 | 6.4734  ± 0.7627 |
| c | 0.0772  ± 0.0031 | 0.0716  ±0.0028 | 0.0528  ± 0.0021 | 0.0773 ±0.0030 | 0.0771  ± 0.0029 | 0.0742  ± 0.0027 | 0.0507  ± 0.0019 | 0.0807  ± 0.0032 | 0.0765  ± 0.0031 | 0.0719  ± 0.0028 | 0.0525  ± 0.0021 | 0.0764  ± 0.0030 |
| **Richards** | |  |  |  |  |  |  |  |  |  |  |  |
| a | 265.0789± 8.2709 | 19.7901  ± 0.8046 | 45.9564  ±12.1186 | 248.4923 ±8.4131 | 242.7192± 5.9384 | 17.8556  ± 0.5453 | 38.7582  ± 5.2721 | 226.1611  ± 6.4712 | 275.6073  ± 9.2243 | 19.1236  ± 0.7372 | 48.9297  ±16.1719 | 252.6581  ± 9.0493 |
| b | 1.9867E-15±1.7776E-05 | 1.74E-15  ±1.6049E-06 | 0.0138  ± 0.0021 | 9.8830E-15 ±6.1073E-06 | 3.6905E-15±1.3271E-05 | 5.3441E-18±9.0445E-07 | 0.0143  ± 0.0014 | 2.5506E-15± 5.6099E-06 | 9.2807E-14±3.2066E-05 | 3.5922E-15±2.8337E-06 | 0.0171  ± 0.0024 | 4.2241E-15  ±1.4407E-05 |
| c | 0.0402  ±0.0028 | 0.0345  ±0.0027 | 0.0021  ± 0.0005 | 0.0431 ±0.0034 | 0.0423  ± 0.0025 | 0.0378  ± 0.0024 | 0.0026  ± 0.0003 | 0.0456  ± 0.0032 | 0.0390  ± 0.0029 | 0.0340  ± 0.0026 | 0.0019  ± 0.0006 | 0.0413  ± 0.0034 |
| n | -0.6244  ± 0.0416 | -0.6021  ± 0.0393 | -0.8650  ± 0.0086 | -0.5807 ±0.0504 | -0.6161  ± 0.0391 | -0.5872  ± 0.0341 | -0.8531  ± 0.0057 | -0.5833  ± 0.0460 | -0.6373  ± 0.0430 | -0.6250  ± 0.0375 | -0.8665  ± 0.0100 | -0.6095  ± 0.0518 |
| **Morgan** |  |  |  |  |  |  |  |  |  |  |  |  |
|  |  |  |  |  |  |  |  |  |  |  |  |  |
| a | 418.8533±12.0810 | 32.9183  ± 1.1353 | 2759.5481±12294.7614 | 410.7723 ±9.2437 | 361.8080±7.7420 | 27.0240  ± 0.6633 | 337.5414  ± 9.1291 | 478.4021  ±108.5936 | 450.9012  ±14.2256 | 31.9903  ± 1.0739 | 3497.6903±31445.1288 | 441.7581  ±10.3218 |
| b | -4.7873  ± 0.5344 | -0.2826  ± 0.0292 | 0.0099  ± 0.0012 | -7.4123 ±0.4646 | -4.5761  ± 0.4768 | -0.2339  ± 0.0248 | -4.6223  ± 0.5480 | 0.0094  ± 0.0004 | -5.1860  ± 0.5647 | -0.2691  ± 0.0283 | 0.0144  ± 0.0018 | -8.0406  ± 0.4581 |
| c | 1.2986  ± 0.0194 | 1.3375  ± 0.0182 | 1.1134  ± 0.0036 | 1.2927 ±0.0155 | 1.3271  ± 0.0175 | 1.3959  ±0.0165 | 1.3632  ± 0.0225 | 1.1221  ± 0.0011 | 1.2667  ± 0.0194 | 1.3054  ± 0.0174 | 1.1127  ± 0.0056 | 1.2294  ± 0.0142 |
| k | 46.2124  ± 1.8170 | 57.5731  ± 2.5172 | 21450.8515±86322.6189 | 47.2380 ±1.4352 | 40.9972  ± 1.2015 | 47.4899  ± 1.4720 | 39.0791  ± 1.4090 | 4236.9910  ± 880.2867 | 49.4518  ± 2.1554 | 58.1186  ± 2.5356 | 27026.8518±219351.8315 | 52.1328  ± 1.7048 |
| 1. Cum. EP = cumulative egg production; Cum, EM = cumulative egg mass; Cum. EW = cumulative egg weight; Cum. Hatchability = cumulative hatching eggs.  2. a, b, c, d = model parameters estimated by nonlinear regression; values are presented as mean ± standard error (SE). | | | | | | | | | | | | |
